# Identification of Species and Subspecies of Lactic Acid Bacteria Present in Spanish Cheeses Type “Torta” by MALDI-TOF MS and *pheS* gene Analyses

**DOI:** 10.3390/microorganisms8020301

**Published:** 2020-02-21

**Authors:** Fernando Sánchez-Juanes, Vanessa Teixeira-Martín, José Manuel González-Buitrago, Encarna Velázquez, José David Flores-Félix

**Affiliations:** 1 Instituto de Investigación Biomédica de Salamanca (IBSAL), Complejo Asistencial Universitario de Salamanca, Universidad de Salamanca, CSIC, 37007 Salamanca, Spain; fsjuanes@gmail.com (F.S.-J.); buitrago@usal.es (J.M.G.-B.); 2 Departamento de Bioquímica y Biología Molecular, Universidad de Salamanca, 37007 Salamanca, Spain; 3 Departamento de Microbiología y Genética and Instituto Hispanoluso de Investigaciones Agrarias (CIALE), Universidad de Salamanca, Edificio Departamental de Biología, Lab 209. Av. Doctores de la Reina S/N, 37007 Salamanca, Spain; vaneteixe23@usal.es; 4 Unidad Asociada Grupo de Interacción Planta-Microorganismo (Universidad de Salamanca-IRNASA-CSIC), 37007 Salamanca, Spain

**Keywords:** lactic acid bacteria, cheese, “Torta” type, MALDI-TOF MS, *pheS* gene, Spain

## Abstract

Several artisanal cheeses are elaborated in European countries, being commonly curdled with rennets of animal origin. However, in some Spanish regions some cheeses of type “Torta” are elaborated using *Cynara cardunculus* L. rennets. Two of these cheeses, “Torta del Casar” and “Torta de Trujillo”, are elaborated in Cáceres province with ewe’s raw milk and matured over at least 60 days without starters. In this work, we identified the lactic acid bacteria present in these cheeses using MALDI-TOF MS and *pheS* gene analyses, which showed they belong to the species *Lactobacillus curvatus*, *Lactobacillus diolivorans*, *Lactobacillus paracasei*, *Lactobacillus plantarum*, *Lactobacillus rhamnosus*, *Lactococcus lactis* and *Leuconostoc mesenteroides*. The *pheS* gene analysis also allowed the identification of the subspecies *La. plantarum* subsp. *plantarum*, *La. paracasei* subsp. *paracasei* and *Le. mesenteroides* subsp. *jonggajibkimchii*. Low similarity values were found in this gene for some currently accepted subspecies of *Lc. lactis* and for the two subspecies of *La. plantarum,* and values near to 100% for the subspecies of *Le. mesenteroides* and *La. paracasei*. These results, which were confirmed by the calculated ANIb and dDDH values of their whole genomes, showed the need to revise the taxonomic status of these species and their subspecies.

## 1. Introduction

Lactic acid bacteria (LAB) encompass Gram positive cocci and rods distributed in different genera, species and subspecies belonging to different families from the order *Lactobacillales* [1]. Many of these bacteria are considered probiotics due to their beneficial effects for human health [2] and they are present in fermented foods [3]. 

Cheeses, including artisanal ones, are commonly curdled with rennet of animal origin, however, the Spanish agronomic writer Columela (4–70 AD) mentioned in his book entitled *De Re Rustica* that cheese can be curdled with the thistle flowers. This practice is currently maintained in some Spanish regions, where the cheeses of type “Torta” are elaborated using *Cynara cardunculus* L. rennets. The best known of these cheeses is the “Torta del Casar” elaborated in Cáceres province with ewe’s raw milk and matured over at least 60 days without starters.

The LAB present in “Torta del Casar” cheese were initially identified using phenotypic traits [4], and more recently through the analysis of the 16S rRNA gene sequences [5], which was the methodology also used for the identification of these bacteria in other European artisanal cheeses [6,7,8,9,10,11]. 

However, the 16S rRNA gene has limitations in differentiating among closely related species and subspecies of LAB needing additional techniques, such as the sequencing of protein-coding genes or MALDI-TOF MS [12]. The latter technique has been used to identify the LAB from a French artisanal cheese, showing the presence of species such as *La. plantarum* and *La. paracasei*, which encompass several subspecies [13]. 

The usefulness of MALDI-TOF MS to differentiate some subspecies of *La. paracasei*, *La. plantarum* and *Lc. lactis* has been shown in some works [14,15,16], but the identification at subspecies level should be assessed by the sequencing of protein-coding genes, which have a higher discriminating power than the 16S rRNA gene among closely related taxa. In the case of LAB, the *pheS* gene has been used, combined with MALDI-TOF MS, for their identification in some fermented foods [17,18], but, to date, these two techniques have not been used together to identify LAB in cheese samples. 

Therefore, the first aim of this work was to identify the LAB isolated from two cheeses of type “Torta” elaborated in two different sites (Casar and Trujillo) in Cáceres province in Spain through MALDI-TOF MS and *pheS* gene analyses. The second aim was to analyse the results obtained with these two techniques compared to those of whole-genome analysis for the differentiation of the subspecies currently accepted within several species of LAB. 

## 2. Materials and Methods 

### 2.1. Strains Isolation 

The strains were isolated from ripened cheeses type “Torta” named “Torta del Casar” (Doña Engracia Torta del Casar, Casar de Cáceres, Spain) and “Torta de Trujillo” (or “Retorta de Trujillo”) (Quesería Finca Pascualete, Trujillo, Spain), both elaborated in Cáceres province. For strains’ isolation, we followed the methodology described by Ordiales et al. [5] using MRS agar (Sigma Co., St. Louis, MO., USA) for strain isolation. The inoculated plates were incubated at 20 °C for 48h. 

### 2.2. MALDI-TOF MS Performing and Data Analysis

The sample preparation and the MALDI-TOF MS analysis were carried out as was previously published [19] using a matrix of saturated solution of α-HCCA (Bruker Daltonics, Bremen, Germany) in 50% acetonitrile and 2.5% trifluoracetic acid. We used amounts of biomass between 5 and 100 mg to obtain the spectra as indicated by the manufacturer. The calibration masses were the Bruker Bacterial Test Standards (BTS), which were as follows (masses as averages): RL36, 4365.3 Da; RS22, 5096.8 Da; RL34, 5381.4 Da; RL33meth, 6255.4 Da; RL29, 7274.5 Da; RS19, 10,300.1 Da; RNase A, 13,683.2 Da and myoglobin, 16,952.3 Da. 

The score values proposed by the manufacturer are the following: a score value between 2.3 and 3.00 indicates highly probable species identification; a score value between 2.0 and 2.299 indicates secure genus identification and probable species identification, a score value between 1.7 and 1.999 indicates probable genus identification, and a score value <1.7 indicates no reliable identification. 

Cluster analysis was performed based on a comparison of strain-specific main spectra, created as described above. The dendrogram was constructed by the statistical toolbox of Matlab 7.1 (MathWorks Inc., Natick, MA, USA) integrated in the MALDI Biotyper 3.0 software. The parameter settings were: ‘Distance Measure=Euclidean’ and ‘Linkage=Complete’. The linkage function is normalized according to the distance between 0 (perfect match) and 1000 (no match).

### 2.3. Phylogenetic Analysis of pheS Gene

The amplification and sequencing of *pheS* gene was carried out as indicated by Doan et al. [17] using the primers pheS-21-F (5’-CAYCCNGCHCGYGAYATGC-3’) and pheS-23-R (5’-GGRTGRACCATVCCNGCHCC-3’). The sequences obtained were compared with those from the GenBank using the BLASTN program [20]. The obtained sequences and those of related bacteria retrieved from GenBank were aligned using the Clustal W program [21]. The phylogenetic distances were calculated according to Kimura´s two-parameter model [22]. The phylogenetic trees were inferred using the neighbour joining model [23] and MEGA 7.09 [24] was used for all the phylogenetic analyses. 

### 2.4. Genome Analysis of the Subspecies from the Species Identified in this Study 

The Average nucleotide identity blast (ANIb) and Digital DNA–DNA hybridization (dDDH) was calculated using the JSpecies service [25] (http://imedea.uib-csic.es/jspecies/) and dDDH values were calculated using the genome-to-genome distance calculator website service from DSMZ (GGDC 2.1) [26] (http://ggdc.dsmz.de/ggdc.php/). These values were calculated using the formula two at the GGDC website because it is the only function appropriate to analyse draft genomes [27].

## 3. Results

### 3.1. MALDI-TOF MS Analysis 

**Figure 1:**
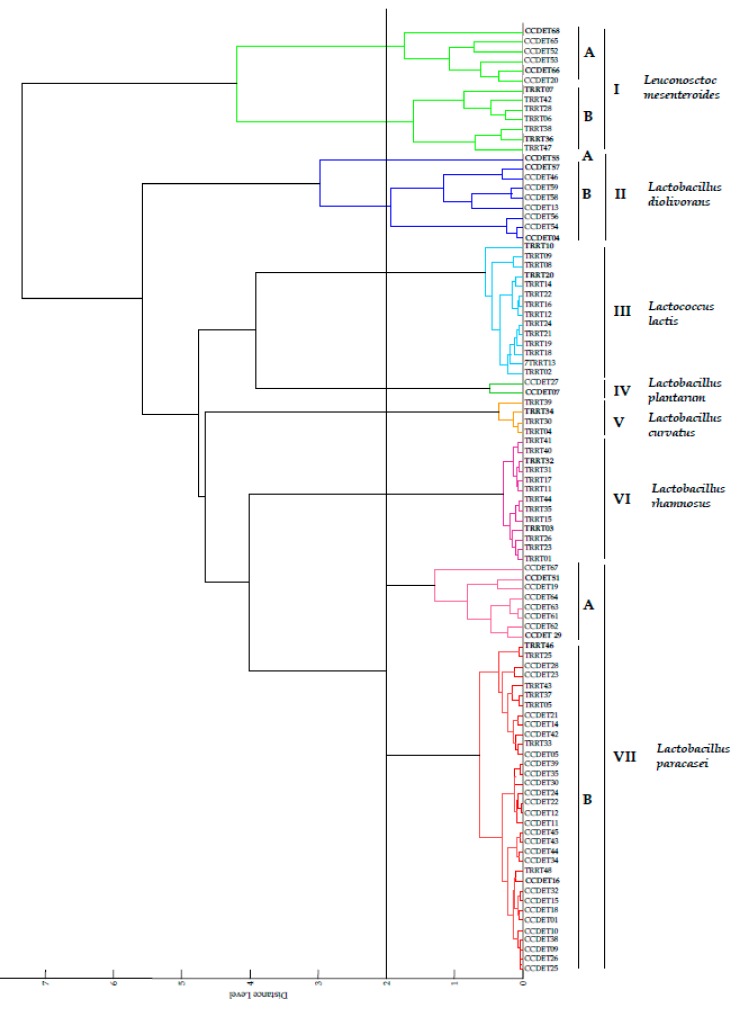
Cluster analysis of MALDI-TOF MS spectra of strains isolated in this study. Distance is displayed in relative units. Representative strains of each group selected for *pheS* gene analysis are marked in bold.

The results of this analysis showed that the isolated strains belong to different genera and species of LAB, namely *La. curvatus*, *La. diolivorans*, *La. paracasei*, *La. plantarum*, *La. rhamnosus*, *Le. mesenteroides* and *Lc. lactis*. All our strains matched with score values near or higher than 2.0 with strains of these species available in the Biotyper 3.0 database (Table 1). Nevertheless, in most cases, the first matching strain is not the strain type of the identified species, and therefore the identification must be confirmed by gene analysis. In order to select representative strains for this analysis, we grouped the isolated strains through mathematical analysis of their, and the resulting dendrogram is shown in Figure 1.

The strains were distributed into seven groups with similarity values lower than 2, which correspond to the different species identified in this study (Figure 1). Group I encompasses strains that matched with score values higher than 2.0 with *Le. mesenteroides* strains and was divided into two subgroups. The strains from the subgroup IA matched with the type strains of *Le. mesenteroides* subsp. *mesenteroides* DSM 20343^T^ and *Le*. *mesenteroides* subsp. *cremoris* DSM 20346^T^ and with the non-type strain of *Le. mesenteroides* subsp. *dextranicum* DSM 20187 with score values lower than 2.3, whereas those from the subgroup IB matched with the type strain of *Le. mesenteroides* subsp. *mesenteroides* DSM 20343^T^ with score values higher than 2.3 (Table 1). 

Group II encompasses strains that matched with the type strain of *La. diolivorans* DSM 14421^T^ and comprised the independent branch IIA and the subgroup IIB (Figure 1). The strain CCDET 55 formed an independent branch and matched with the type strain of *La. diolivorans* DSM 14421^T^ with a score value lower than 2.0, whereas the strains from subgroup IIB matched with score values higher than 2.0 and lower than 2.3 with the same type strain (Table 1). 

Group III encompasses strains that matched with score values higher than 2.0 with *Lc. lactis* strains (Figure 1). All strains isolated in this study matched with the non-type strain *Lc. lactis* subsp. *lactis* DSM 20661, with score values near to or higher than 2.3 with *Lc. lactis* subsp. *lactis* DSM 20481^T^ with score values lower than 2.3 and with *Lc*. *lactis* subsp. *cremoris* DSM 20069^T^ with score values lower than 2.0 in most of cases (Table 1). 

Group IV encompasses two strains that matched with score values higher than 2.0 with *La. plantarum* strains (Figure 1). The strain CCDET07 matched with score values higher than 2.3 with the non type strain *La. plantarum* DSM 2601 and with the type strain of *La. plantarum* subsp. *argentoratensis* DSM 16365^T^, whereas these values were lower than 2.3 with respect to the type strain of *La. plantarum* subsp. *plantarum* DSM 20174^T^. The strain CCDET27 matched with score values higher than 2.0 with respect to the non-type strain *La. plantarum* DSM 12028 and with the type strain of *La. plantarum* subsp. *argentoratensis* DSM 16365^T^, whereas these values were lower than 2.0 with respect to the type strain of *La. plantarum* subsp. *plantarum* DSM 20174^T^ (Table 1). 

Group V encompasses strains matching with score values higher than 2.0 with *La. curvatus* strains (Figure 1). The higher score values, near or higher than 2.3, were found with respect to the non-type strain DSM 20499, whereas these values were lower than 2.3 with respect to the type strain of *La. curvatus* DSM 20499^T^ (Table 1). 

Group VI encompasses strains that matched with the type strain of *La. rhamnosus* CIP A157^T^ with score values higher than 2.3 in all cases (Figure 1, Table 1). 

Finally, group VII encompasses strains that matched with score values higher than 2.0 with *La. paracasei* strains (Figure 1). This group was divided into two subgroups whose strains mostly matched with score values higher than 2.3 with different non-type strains of *La. paracasei* subsp. *paracasei* (DSM 20006, DSM 20244, DSM 2649, DSM 20312 or DSM 8741). Only the strain CCDET19 matched with values higher than 2.3 with respect to the type strain of *La. paracasei* subsp. *tolerans* DSM 20258^T^ and the remaining strains matched with *La. paracasei* subsp. *paracasei* DSM 5622^T^ and/or *La. paracasei* subsp. *tolerans* DSM 20258^T^ with score values lower, near or higher than 2.0, but in all cases lower than 2.3 (Table 1). 

Since the type strains of several subspecies identified in this study are included in the Biotyper 3.0 database, we calculated the score values between the subspecies from the same species (Table 2). Score values higher than 2.3, typically found in strains from the same species, were presented by the type strains of the subspecies *plantarum* and *argentoratensis* of *La. plantarum* (2.424) and by those of the subspecies *mesenteroides* and *cremoris* of *Le. mesenteroides* (2.456). However, score values lower than 2.3, which can be found in strains of different species, were found between by the type strains of the subspecies *lactis* and *cremoris* of *Lc. lactis* (2.174) and by those of the subspecies *paracasei* and *tolerans* of *La. paracasei* (1.846). These results show the need to carry out genetic analyses to verify the taxonomic status of these subspecies.

### 3.2. pheS Gene Analysis 

**Figure 2:**
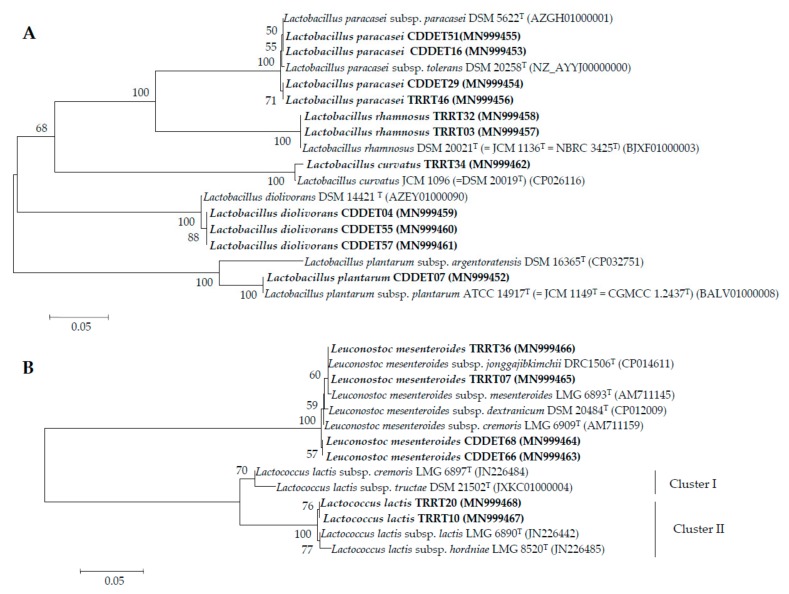
(**A**) Neighbour-joining phylogenetic unrooted tree based on *pheS* gene partial sequences (400 nt) showing the taxonomic location of representative strains from different groups of MALDI-TOF MS within the genus *Lactobacillus*. (**B**) Neighbour-joining phylogenetic unrooted tree based on *pheS* gene partial sequences (400 nt) showing the taxonomic location of representative strains from different groups of MALDI-TOF MS within the genera *Lactobacillus* and *Leuconostoc*. Bootstrap values calculated for 1000 replications are indicated. Bar, 5 nt substitution per 1000 nt. Accession numbers from Genbank are given in brackets.

The analysis of partial sequences of *pheS* gene of representative strains of different MALDI-TOF MS groups are shown in Figure 2 and Table 2 and Table 3. The results of this analysis confirmed the identification obtained after MALDI-TOF MS analysis at genus and species levels for all strains isolated in this study. 

According to the results of the *pheS* gene analysis, several strains were identified with high similarity values as *Lactobacillus* species that, to date, do not encompasses subspecies (Figure 2A, Table 3). The strains TRRT03, TRRT32 representative of group VI, were identified as *La. rhamnosus* with 100% similarity. The strain CCDET55, representative of subgroup IIA, and the strains CCDET04, CCDET57, representative of subgroup IIB, were identified as *La. diolivorans* with 99.3% similarity. The strain TRRT34, representative of group V, was identified as *La. curvatus* with 99.2% similarity.

After the *pheS* gene analysis, the remaining strains were identified with high similarity values with LABs of species that contain two or more subspecies (Figure 2A, Table 3). This happened in the case of the strain CCDET07, representative of group IV, which was identified as *La. plantarum* which currently encompasses two subspecies, *L. plantarum* subsp. *plantarum* and *L. plantarum* subsp. *argentoratensis*, whose type strains showed 90.5% similarity in their *pheS* gene sequences (Table 2). The strain CCDET07, representative of group II, can be assigned to the subspecies *plantarum,* since it presented 100% similarity with respect to the type strain of this subspecies and 90.5% similarity with respect to the type strain of the subspecies *argentoratensis* (Figure 2B, Table 3). 

The representative strains from group I were identified with *pheS* gene similarity values higher than 99.2% with the species *Le. mesenteroides*, whose subspecies *mesenteroides*, *cremoris*, *dextranicum* and *jonggajibkimchii* showed values ranging from 99.2% to 99.7% (Figure 2B, Table 2). The strains CCDET66, CCDET68 representative of subgroup IA were slightly more closesly related to the type strain of *Le. mesenteroides* subsp. *cremoris,* with 99.7% similarity, than to the type strains of the remaining subspecies, with similarity values ranging from 99.2% to 99.5%. The strains TRRT07, TRRT36, representative of subgroup IB, presented 100% similarity with respect to the type strain of *Le. mesenteroides* subsp. *jonggajibkimchii*, and values ranging from 99.5% to 99.7% with respect to the type strains of the other three subspecies. Therefore, the strains from the group IB can be assigned to the subspecies *Le. mesenteroides* subsp. *jonggajibkimchii,* whereas it is difficult to assign those of group IA to any of the subspecies from *Le. mesenteroides* (Figure 2B, Table 3).

The representative strains from group III were identified with *pheS* gene similarity values higher than 99.0% as *Lc. lactis*, whose subspecies formed two clearly separated clusters with less than 93% similarity (Figure 2B, Table 2). Cluster I contains the subspecies *lactis* and *hordniae* showing 99.2% similarity and cluster II the subspecies *cremoris* and *tructae,* showing 98.5% similarity (Table 2). The strains TRRT10, TRRT20, representative of group III, belong to cluster II and, since they presented 99.0% and 99.5% similarity, respectively, to the subspecies *lactis* and *hordniae,* it is difficult to assign the strains of group III to any of these two subspecies (Figure 2B, Table 3). 

The representative strains from group VII with *pheS* gene similarity values higher than 99.5% were identified as *La. paracasei*, which contains two subspecies, *paracasei* and *tolerans*, showing 99.5% similarity between their type strains (Figure 2A, Table 2). The representative strains for both subgroups VIIA and VIIB were divided into two subclusters with 99.5% similarity, each one containing strains of these both subgroups (Figure 2A). The strains CCDET51 and CCDET16 can be assigned to the subspecies *paracasei* since they showed 100% similarity with the type strain of this subspecies, however, the strains CCDET29 and CCDET46 cannot be assigned to these subspecies because they showed 99.5% similarity with respect to their type strains (Figure 2A, Table 3).

Therefore, the identification at species level obtained by MALDI-TOF MS was confirmed by *pheS* gene sequencing. Moreover, the *pheS* gene analysis supports the identification at subspecies level for some strains isolated in this work, but it is remarkable that several others cannot be assigned to any subspecies because they formed subclusters whose similarity values are similar to those found among the currently accepted subspecies of LAB identified in this study.

**Figure 3:**
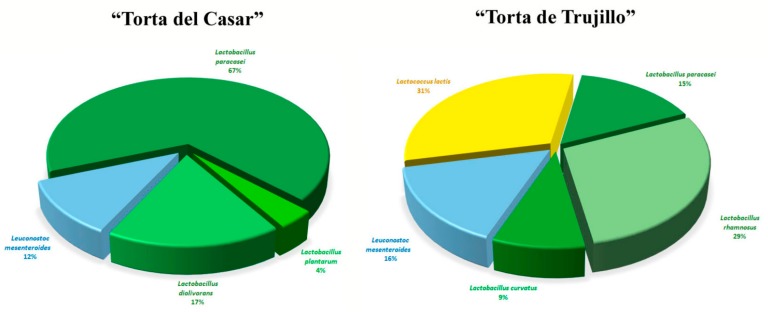
Pie charts showing the distribution of the different species of LAB in the two cheeses type “Torta” analysed in this study.

Collectively, the data from MALDI-TOF MS and *pheS* gene analyses showed that most of the strains isolated from “Torta del Casar” belong to the species *La. paracasei,* which was also present in “Torta de Trujillo” and that the species *Le. mesenteroides* was present in both cheeses in similar proportions. However, other species only were found in one of the two cheeses, *La. diolivorans* and *La. plantarum* in “Torta del Casar”, and *La. curvatus*, *La. rhamnosus* and *Lc. lactis* in “Torta de Trujillo” (Figure 3, Table 2). 

### 3.3. Taxonomic Status of the Subspecies from the Species Identified in this Study 

The *pheS* gene analysis showed that similarity values ranging from 98.5% to 99.7% are presented by the type strains of the subspecies of *La. paracasei* and *Le. mesenteroides*, whereas values lower than 93% were found between the type strains of the subspecies of *La. plantarum* and those of some subspecies of *Lc. lactis* (Table 2). These results should be compared with those obtained after whole genome analysis, taking into account the threshold values of ANIb and dDDH for bacterial species differentiation (95%~96% and 70%, respectively) [28] and the dDDH cut-off values for bacterial subspecies differentiation (79%~80%) [29].

The whole genomes of the type strains of all subspecies found in this study are available in Genbank and we calculated the ANIb and dDDH values for all of them, whether or not they are present in the Biotyper 3.0 database (Table 2). In agreement with the results of both *pheS* gene and MALDI-TOF MS analyses, the type strains of the subspecies *mesenteroides* and *cremoris* of *Le. mesenteroides* showed ANIb and dDDH values typical of the same species, 98.1% and 90.9%, respectively (Table 2). Concerning the subspecies *dextranicum* and *jonggajibkimchii*, whose type strains are not in Biotyper 3.0 database, in agreement with the results of *pheS* gene analysis, their ANIb and dDDH values, between them and with respect to the remaining two subspecies, were higher than those proposed for bacterial species differentiation (Table 2). 

In agreement with the results of both *pheS* gene and MALDI-TOF MS analyses, the type strains of the subspecies *lactis* and *cremoris* of *Lc. lactis* showed ANIb and dDDH values typical of different species, 86.7% and 33.1 %, respectively (Table 2). These results confirmed that the type strains of the subspecies *cremoris* and *lactis* belong to different species, making it necessary to reclassify the subspecies *cremoris* into a different, novel species. Nevertheless, it is also necessary to analyse the two subspecies of *Lc. lactis* that are not present in the Biotyper 3.0 database, as the *pheS* gene analysis showed that they belong to two divergent clusters, one of them containing the type strains of the subspecies *lactis* and *hordniae* and the other containing the type strains of the subspecies *cremoris* and *tructae*. Taking into account the ANIb and dDDH values found among the type strains of these subspecies, the subspecies *hordniae* should be maintained within the species *Lc. lactis,* and the subspecies *tructae,* together with the subspecies *cremoris,* should be transferred to a novel species (Table 2). 

In agreement with the results of the *pheS* gene analysis, but not with those of MALDI-TOF MS analysis, the type strains of the subspecies *plantarum* and *argentoratensis* of *La. plantarum*, which showed ANIb and dDDH values typical of different species, 94.9% and 62.9%, respectively, should be considered different species, making it necessary to reclassify the subspecies *argentoratensis* in a novel species. In agreement with the results of the *pheS* gene analysis, but not with those of MALDI-TOF MS analysis, the type strains of the subspecies *paracasei* and *tolerans* of *La. paracasei* showed ANIb and dDDH values typical of the same species, 97.9% and 84.9%, respectively (Table 2). 

## 4. Discussion

There is a growing interest in the identification of LAB present in artisanal cheeses elaborated with raw milk in Europe, with those elaborated with cow or/and goat raw milks being more analysed [6,7,8,10,11,13] compared to those elaborated with ewe’s raw milk [5,9]. 

In Spain, one of the most appreciated cheeses is the named type “Torta”, elaborated with ewe’s raw milk in Caceres province, and therefore it is also interesting to know the species of LAB present in these cheeses. In a work published in the last century, the lactic bacteria present in the “Torta del Casar” cheese were identified on the basis of phenotypic traits [4]. More recently, by 16S rRNA gene analysis, the species *Lactobacillus sakei*, *Lactobacillus casei*, *Lactobacillus helveticus* and *Lc. lactis* subsp. *cremoris* have been identified in this cheese [5]. These two works were only carried out in the “Torta del Casar” cheese and using techniques that have limitations for species and particularly for subspecies differentiation. For this reason, in this study we compared the results obtained in two cheeses type “Torta” elaborated in the same region by using more recent methodologies. From the species previously identified in the “Torta del Casar” cheese [5], in the present work only *L. lactis* has been identified in the “Torta de Trujillo” cheese. Nevertheless, the species identified in this study have been found in some of the European artisanal cheeses elaborated with raw milk [7,8,9,10,11,12,13].

From the mentioned cheeses, only the LAB present in the French cheese Maroilles were identified by MALDI-TOF MS [13]. The authors showed that this methodology is very useful to identify LAB belonging to different genera and species, but they do not demonstrate its usefulness in differentiating among subspecies. Considering that many species of LAB contain several subspecies, this is an essential issue to be discussed by comparison with other molecular techniques, particularly genomic ones. 

**Figure 4:**
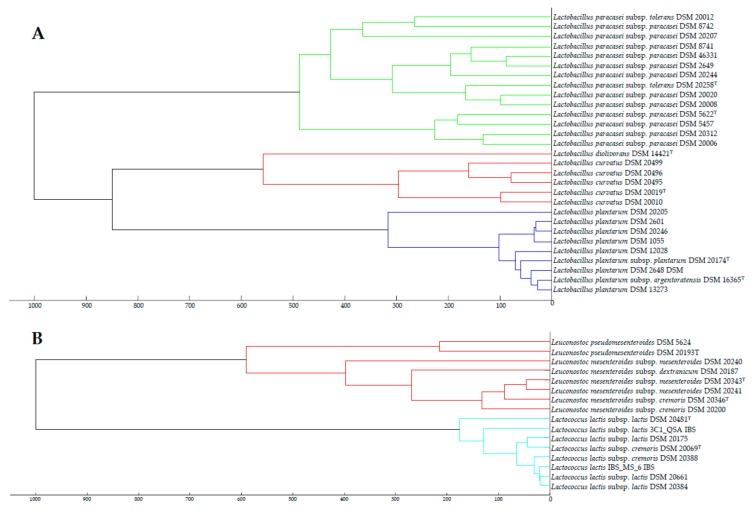
Cluster analysis of MALDI-TOF MS spectra of the strains belonging to the species identified in this study which are included in the biotyper 3.0 database within genera *Lactobacillus* (**A**) and *Leuconostoc* and *Lactococcus* (**B**). Distance is displayed in relative units.

In this study, we identified four species which contain several subspecies, *La. plantarum*, *La. paracasei*, *Le. mesenteroides* and *Lc. lactis* (http://www.bacterio.net/) of which the most common inhabitants in milk-related sources are present in the database Biotyper 3.0. In addition to the type strain, several strains are included in this database for *Le. mesenteroides* subsp. *mesenteroides*, *Lc. lactis* subsp. *lactis*, *Lc. lactis* subsp. *cremoris*, *La. paracasei* subsp. *paracasei*, *La. paracasei* subsp. *tolerans* and *La. plantarum* subsp. *plantarum*. The presence of most than one strain for a taxon in a database is *a priori* positive, but this can be an important disadvantage if some strains are not correctly assigned to a taxon, as seems to occur for several strains from the subspecies of *Le. mesenteroides*, *Lc. lactis*, *La. paracasei* and *La. Plantarum,* present in the Biotyper 3.0 database. For example, the non-type strain of *La. plantarum* subsp. *plantarum* DSM 20205 is more distant from the type strain of this subspecies than the type strain of *La. plantarum* subsp. *argentoratensis* (Figure 4A). In the case of *La. paracasei,* there is a greater distance among strains of the same subspecies than among strains of different species (Figure 4A). In the case of *Le. mesenteroides*, the non-type strain *Le*. *mesenteroides* subsp. *mesenteroides* DSM 2040 is more distant from the type strain of this subspecies than the strain *Le. mesenteroides* subsp. *dextranicum* DSM 20187 (Figure 4B). Several strains assigned to *Lc. lactis* subsp. *lactis* are more distant from the type strain of this subspecies than to that of *Lc. lactis* subsp. *cremoris* (Figure 4B). These results indicate that several non-type strains held in DSMZ culture collection which are included in the Biotyper 3.0 database are not correctly classified at species or subspecies levels, but, as no gene sequences are available for these strains, we cannot know their correct taxonomic name and this could lead to errors in the identification of any tested strain. For this reason, we always referred to a type strain in the identification of our strains, although the score values were lower than those found for non-type strains (Table 1). 

Moreover, we found some surprising score values for the type strains of the subspecies from *La. paracasei* and *Lc. lactis* because the subspecies are infraspecific taxa and score values higher than 2.3 among these subspecies are expected after MALDI-TOF MS analysis (Table 2). In the case of the type strains of the subspecies *paracasei* and *tolerans* of *La. paracasei,* the score value was clearly lower than 2.0, indicating that these strains do not belong to the same species (Table 2). This contrasts with the high similarity value of the *pheS* gene sequences and the high ANIb and dDDH values calculated from their genomes (Table 2). These two values, which are clearly higher than those proposed for species differentiation [28], confirmed that the type strains of *paracasei* and *tolerans* belong to the same species, therefore the type strains of these subspecies held in DSMZ culture collection and in the Biotyper 3.0 database should be revised. In addition, they showed that the dDDH values are higher than those proposed for subspecies differentiation [29], therefore the taxonomic status of these subspecies should be revised.

In the case of the type strains of the subspecies *lactis* and *cremoris* of *Lc. lactis*, the low values found in the *pheS* gene analysis agree with the calculated ANIb and dDDH values, which were lower than those proposed for species differentiation, confirming that they belong to different species (Table 2). Concerning to the other two subspecies, *hordniae* and *tructae*, not included in Biotyper database, the *pheS* gene analysis showed that their type strains are phylogenetically related to the subspecies *lactis* and *cremoris*, respectively (Table 2). The calculated ANIb and dDDH values confirmed that *Lc. lactis* really contains two different species with two subspecies each, although the dDDH values were near to or slightly lower than those proposed for subspecies differentiation in both cases (Table 2). These results clearly indicate that the taxonomic status of the subspecies currently included within *Lc. lactis* should be revised in order to separate the subspecies *cremoris* as a novel species and to evaluate whether the subspecies *hordniae* and *tructae* can maintain their current taxonomic status.

Conversely, the score value found between the type strains of the subspecies *plantarum* and *argentoratensis* of *La. plantarum* was surprisingly high (2.424) considering the low similarity value found between their *pheS* genes (90.5%), and that the calculated ANIb and dDDH values were lower than those proposed for bacterial species differentiation (Table 2). These results indicate that the type strain of the subspecies *argentoratensis* held in DSMZ culture collection and in the Biotyper 3.0 database should be revised and that it should be reclassified as a novel species.

In the case of the subspecies *mesenteroides* and *cremoris* of *Le. mesenteroides* score, values higher than 2.3 were expected, as this corresponds to strains from the same species (Table 2). In agreement, they showed high similarity in their *pheS* genes and the calculated ANIb and dDDH values were higher than those proposed for bacterial species differentiation (Table 2). In the case of the subspecies *dextranicum* and *jonggajibkimchii*, absent in the Biotyper 3.0 database, the *pheS* gene analysis also showed high similarity values in agreement with those of the ANIb and dDDH, which were also higher than those proposed for bacterial species’ differentiation. Moreover, the dDDH values among the type strains of all these subspecies considerably exceed the upper limit proposed for subspecies differentiation, and therefore their taxonomic status should be revised. 

Therefore, the application of the currently accepted ANIb and dDDH cut-off values for species differentiation [28] will lead to the promotion of some subspecies to the taxonomic status of species. Conversely, the application of the dDDH threshold value for subspecies differentiation [29] will lead to the loss of taxonomic status for some subspecies, as recently occurred with the subspecies *sakuensis* of *Serratia marcescens* [30]. Although Chun et al. [28] considered that currently there is not enough information to establish general guidelines for species differentiation on the basis of genome data, we face the dilemma of whether to increase the dDDH threshold value for subspecies differentiation, maintain the existing ones, or reject many of the existing subspecies in several species of LAB. Before trying to solve this, we should take into account that the increase necessary to maintain the current subspecies would cause a dramatic increase in the number of these taxa, since the *pheS* gene similarity cut-off values would be above 99% and only in this study several strains cannot be assigned to any of the described subspecies because they fall within these limits and could be considered as novel subspecies. We should also consider that applying the dDDH thresholds values proposed by Meier-Kolthoff et al. [29] would mean that none of the subspecies from the species identified in this study can maintain their taxonomic status, except perhaps the subspecies *hordniae* of *Lc*. *lactis*. This second option better agrees with the results of the MALDI-TOF MS analysis that clearly allows the identification of the strains isolated at species level, compared to identification at subspecies level. In any case, this situation should be clarified, since it affects several LABs from different genera and species, and currently there is an increasing interest in the identification of these bacteria, particularly in fermented foods.

## 5. Conclusions

The LAB present in the two cheeses of type “Torta” analysed in this study were identified as *La. curvatus*, *La. diolivorans*, *La. paracasei*, *La. plantarum*, *La. rhamnosus*, *Lc. lactis* and *Le. mesenteroides* through MALDI-TOF MS and *pheS* gene analyses. These results confirmed that MALDI-TOF MS is a reliable method for the identification of LAB comparable to *pheS* gene sequence analysis and presents important advantages over gene sequencing in terms of rapidity and cost per sample. The analysis of *pheS* gene showed low similarity values for some subspecies of *Lc. lactis* and for the two subspecies of *La.* plantarum and values near to 100% for the subspecies of *Le. mesenteroides* and *La. paracasei*. These results were confirmed by the calculated ANIb and dDDH values of their whole genomes, showing the need for a revision of the taxonomic status of these species and their subspecies, which should be based on additional criteria.

## Figures and Tables

**Table 1:** Results obtained using MALDI-TOF MS analysis.

Torta del Casar			
Strains	Closest Taxa	Score Values	Groups
CCDET 01	*La. paracasei* subsp. *paracasei* DSM 20006	2.502	VIIB
	*La. paracasei* subsp. *paracasei* DSM 5622^T^	2.194	
	*La. paracasei* subsp. *tolerans* DSM 20258^T^	1.960	
CCDET 04	*La. diolivorans* DSM 14421^T^	2.228	IIB
CCDET 05	*La. paracasei* subsp. *paracasei* DSM 20006	2.504	VIIB
	*La. paracasei* subsp. *paracasei* DSM 5622^T^	2.193	
	*La. paracasei* subsp. *tolerans* DSM 20258^T^	2.174	
CCDET 07	*La. plantarum* DSM 2601	2.478	IV
	*La. plantarum* subsp. *argentoratensis* DSM 16365^T^	2.322	
	*La. plantarum* subsp. *plantarum* DSM 20174^T^	2.037	
CCDET 09	*La. paracasei* subsp. *paracasei* DSM 20006	2.511	VIIB
	*La. paracasei* subsp. *paracasei* DSM 5622^T^	2.128	
	*La. paracasei* subsp. *tolerans* DSM 20258^T^	1.476	
CCDET 10	*La. paracasei* subsp. *paracasei* DSM 20006	2.483	VIIB
	*La. paracasei* subsp. *paracasei* DSM 5622^T^	2.097	
	*La. paracasei* subsp. *tolerans* DSM 20258^T^	2.051	
CCDET 11	*La. paracasei* subsp. *paracasei* DSM 20244	2.517	VIIB
	*La. paracasei* subsp. *paracasei* DSM 5622^T^	2.063	
	*La. paracasei* subsp. *tolerans* DSM 20258^T^	1.911	
CCDET 12	*La. paracasei* subsp. *paracasei* DSM 20006	2.433	VIIB
	*La. paracasei* subsp. *tolerans* DSM 20258^T^	2.113	
	*La. paracasei* subsp. *paracasei* DSM 5622^T^	2.018	
CCDET 13	*La. diolivorans* DSM 14421^T^	2.218	IIB
CCDET 14	*La. paracasei* subsp. *paracasei* DSM 2649	2.43	VIIB
	*La. paracasei* subsp. *paracasei* DSM 5622^T^	2.047	
	*La. paracasei* subsp. *tolerans* DSM 20258^T^	1.773	
CCDET 15	*La. paracasei* subsp. *paracasei* DSM 20006	2.531	VIIB
	*La. paracasei* subsp. *paracasei* DSM 5622^T^	2.053	
	*La. paracasei* subsp. *tolerans* DSM 20258^T^	1.542	
CCDET 16	*La. paracasei* subsp. *paracasei* DSM 20006	2.545	VIIB
	*La. paracasei* subsp. *tolerans* DSM 20258^T^	2.147	
	*La. paracasei* subsp. *paracasei* DSM 5622^T^	2.112	
CCDET 18	*La. paracasei* subsp. *paracasei* DSM 20006	2.463	VIIB
	*La. paracasei* subsp. *paracasei* DSM 5622^T^	2.107	
	*La. paracasei* subsp. *tolerans* DSM 20258^T^	1.911	
CCDET19	*La. paracasei* subsp. *paracasei* DSM 20244	2.500	VIIA
	*La. paracasei* subsp. *tolerans* DSM 20258^T^	2.309	
	*La. paracasei* subsp. *paracasei* DSM 5622^T^	2.103	
CCDET20	*Le. mesenteroides* subsp. *dextranicum* DSM 20187	2.062	IA
	*Le. mesenteroides* subsp. *mesenteroides* DSM 20343^T^	1.683	
	*Le. mesenteroides* subsp. *cremoris* DSM 20346^T^	1.648	
CCDET21	*La. paracasei* subsp. *paracasei* DSM 20006	2.337	VIIB
	*La. paracasei* subsp. *tolerans* DSM 20258^T^	2.174	
	*La. paracasei* subsp. *paracasei* DSM 5622^T^	1.952	
CCDET 22	*La. paracasei* subsp. *paracasei* DSM 20006	2.380	VIIB
	*La. paracasei* subsp. *paracasei* DSM 5622^T^*La. paracasei* subsp. *tolerans* DSM 20258^T^	2.0401.752	
CCDET 23	*La. paracasei* subsp. *paracasei* DSM 20244	2.397	VIIB
	*La. paracasei* subsp. *paracasei* DSM 5622^T^	1.938	
	*La. paracasei* subsp. *tolerans* DSM 20258^T^	1.800	
CCDET 24	*La. paracasei* subsp. *paracasei* DSM 20312	2.355	VIIB
	*La. paracasei* subsp. *paracasei* DSM 5622^T^	2.038	
	*La. paracasei* subsp. *tolerans* DSM 20258^T^	1.897	
CCDET 25	*La. paracasei* subsp. *paracasei* DSM 20006	2.544	VIIB
	*La. paracasei* subsp. *paracasei* DSM 5622^T^	2.115	
	*La. paracasei* subsp. *tolerans* DSM 20258^T^	2.092	
CCDET 26	*La. paracasei* subsp. *paracasei* DSM 20006	2.476	VIIB
	*La. paracasei* subsp. *paracasei* DSM 5622^T^	2.165	
	*La. paracasei* subsp. *tolerans* DSM 20258^T^	2.033	
CCDET 27	*La. plantarum* subsp. *plantarum* DSM 12028	2.177	IV
	*La. plantarum* subsp. *argentoratensis* DSM 16365^T^	2.131	
	*La. plantarum* subsp. *plantarum* DSM 20174^T^	1.963	
CCDET 28	*La. paracasei* subsp. *paracasei* DSM 20244	2.386	VIIB
	*La. paracasei* subsp. *tolerans* DSM 20258^T^	2.097	
	*La. paracasei* subsp. *paracasei* DSM 5622^T^	2.097	
CCDET 29	*La. paracasei* subsp. *paracasei* DSM 46331	2.432	VIIA
	*La. paracasei* subsp. *tolerans* DSM 20258^T^	2.224	
	*La. paracasei* subsp. *paracasei* DSM 5622^T^	2.072	
CCDET 30	*La. paracasei* subsp. *paracasei* DSM 20006	2.492	VIIB
	*La. paracasei* subsp. *paracasei* DSM 5622^T^	2.157	
	*La. paracasei* subsp. *tolerans* DSM 20258^T^	2.003	
CCDET 32	*La. paracasei* subsp. *paracasei* DSM 20006	2.513	VIIB
	*La. paracasei* subsp. *paracasei* DSM 5622^T^	2.113	
	*La. paracasei* subsp. *tolerans* DSM 20258^T^	1.540	
CCDET 34	*La. paracasei* subsp. *paracasei* DSM 20244	2.444	VIIB
	*La. paracasei* subsp. *tolerans* DSM 20258^T^	2.160	
	*La. paracasei* subsp. *paracasei* DSM 5622^T^	2.120	
CCDET 35	*La. paracasei* subsp. *paracasei* DSM 20006	2.475	VIIB
	*La. paracasei* subsp. *paracasei* DSM 5622^T^	2.080	
	*La. paracasei* subsp. *tolerans* DSM 20258^T^	1.962	
CCDET 38	*La. paracasei* subsp. *paracasei* DSM 20006	2.452	VIIB
	*La. paracasei* subsp. *paracasei* DSM 5622^T^	2.112	
	*La. paracasei* subsp. *tolerans* DSM 20258^T^	2.083	
CCDET 39	*La. paracasei* subsp. *paracasei* DSM 20006	2.494	VIIB
	*La. paracasei* subsp. *paracasei* DSM 5622^T^	2.059	
	*La. paracasei* subsp. *tolerans* DSM 20258^T^	1.847	
CCDET 42	*La. paracasei* subsp. *paracasei* DSM 20006	2.223	VIIB
	*La. paracasei* subsp. *paracasei* DSM 5622^T^	1.871	
	*La. paracasei* subsp. *tolerans* DSM 20258^T^	1.854	
CCDET 43	*La. paracasei* subsp. *paracasei* DSM 20244	2.348	VIIB
	*La. paracasei* subsp. *paracasei* DSM 5622^T^	2.035	
	*La. paracasei* subsp. *tolerans* DSM 20258^T^	1.990	
CCDET 44	*La. paracasei* subsp. *paracasei* DSM 20244	2.339	VIIB
	*La. paracasei* subsp. *tolerans* DSM 20258^T^	2.106	
	*La. paracasei* subsp. *paracasei* DSM 5622^T^	1.998	
CCDET 45	*La. paracasei* subsp. *paracasei* DSM 20244	2.353	VIIB
	*La. paracasei* subsp. *paracasei* DSM 5622^T^	2.061	
	*La. paracasei* subsp. *tolerans* DSM 20258^T^	2.053	
CCDET 46	*La. diolivorans* DSM 14421^T^	2.235	IIB
CCDET51	*La. paracasei* subsp. *paracasei* DSM 20244	2.437	VIIA
	*La. paracasei* subsp. *tolerans* DSM 20258^T^	2.054	
	*La. paracasei* subsp. *paracasei* DSM 5622^T^	2.054	
CCDET52	*Le. mesenteroides* subsp. *dextranicum* DSM 20187	2.000	IA
	*Le. mesenteroides* subsp. *cremoris* DSM 20346^T^	1.690	
	*Le. mesenteroides* subsp. *mesenteroides* DSM 20343^T^	1.374	
CCDET53	*Le. mesenteroides* subsp. *mesenteroides* DSM 20241	2.120	IA
	*Le. mesenteroides* subsp. *mesenteroides* DSM 20343^T^	2.106	
	*Le. mesenteroides* subsp. *cremoris* DSM 20346^T^	1.961	
CCDET 54	*La. diolivorans* DSM 14421^T^	2.003	IIB
CCDET 55	*La. diolivorans* DSM 14421^T^	1.911	IIB
CCDET 56	*La. diolivorans* DSM 14421^T^	2.093	IIB
CCDET 57	*La. diolivorans* DSM 14421^T^	2.100	IIB
CCDET 58	*La. diolivorans* DSM 14421^T^	2.149	IIB
CCDET 59	*La. diolivorans* DSM 14421^T^	2.106	IIB
CCDET 61	*La. paracasei* subsp. *paracasei* DSM 20006	2.353	VIIA
	*La. paracasei* subsp. *paracasei* DSM 5622^T^	2.170	
	*La. paracasei* subsp. *tolerans* DSM 20258^T^	2.101	
CCDET62	*La. paracasei* subsp. *paracasei* DSM 20244	2.536	VIIA
	*La. paracasei* subsp. *tolerans* DSM 20258^T^	2.157	
	*La. paracasei* subsp. *paracasei* DSM 5622^T^	2.148	
CCDET 63	*La. paracasei* subsp. *paracasei* DSM 8741	2.383	VIIA
	*La. paracasei* subsp. *paracasei* DSM 5622^T^	2.100	
	*La. paracasei* subsp. *tolerans* DSM 20258^T^	2.082	
CCDET64	*La. paracasei* subsp. *paracasei* DSM 20244	2.493	VIIA
	*La. paracasei* subsp. *tolerans* DSM 20258^T^	2.149	
	*La. paracasei* subsp. *paracasei* DSM 5622^T^	2.092	
CCDET65	*Le. mesenteroides* subsp. *dextranicum* DSM 20187	2.072	IA
	*Le. mesenteroides* subsp. *cremoris* DSM 20346^T^	1.692	
	*Le. mesenteroides* subsp. *mesenteroides* DSM 20343^T^	1.454	
CCDET66	*Le. mesenteroides* subsp. *dextranicum* DSM 20187	2.071	IA
	*Le. mesenteroides* subsp. *cremoris* DSM 20346^T^	1.633	
	*Le. mesenteroides* subsp. *mesenteroides* DSM 20343^T^	1.355	
CCDET67	*La. paracasei* subsp. *paracasei* DSM 20244	2.468	VIIA
	*La. paracasei* subsp. *tolerans* DSM 20258^T^	2.233	
	*La. paracasei* subsp. *paracasei* DSM 5622^T^	2.047	
CCDET68	*Le. mesenteroides* subsp. *mesenteroides* DSM 20343^T^	2.204	IA
	*Le. mesenteroides* subsp. *dextranicum* DSM 20187	2.089	
	*Le. mesenteroides* subsp. *cremoris* DSM 20346^T^	1.953	
**Torta de Trujillo**			
**Strains**	**Closest taxa**	**Score values**	**Groups**
TRRT01	*La. rhamnosus* CIP A157^T^	2.362	VI
TRRT02	*Lc. lactis* subsp. *lactis* DSM 20661	2.433	III
	*Lc. lactis* subsp. *lactis* DSM 20481^T^	2.149	
	*Lc. lactis* subsp. *cremoris* DSM 20069^T^	1.913	
TRRT03	*La. rhamnosus* CIP A157^T^	2.389	VI
TRRT04	*La. curvatus* DSM 20499	2.430	V
	*La. curvatus* DSM 20019^T^	2.007	
TRRT05	*La. paracasei* subsp. *paracasei* DSM 20006	2.393	VIIB
	*La. paracasei* subsp. *tolerans* DSM 20258^T^	2.193	
	*La. paracasei* subsp. *paracasei* DSM 5622^T^	2.109	
TRRT06	*Le. mesenteroides* subsp. *mesenteroides* DSM 20343^T^	2.368	IB
	*Le. mesenteroides* subsp. *dextranicum* DSM 20187	2.097	
	*Le. mesenteroides* subsp. *cremoris* DSM 20346^T^	1.963	
TRRT07	*Le. mesenteroides* subsp. *mesenteroides* DSM 20343^T^	2.380	IB
	*Le. mesenteroides* subsp. *cremoris* DSM 20346^T^	2.221	
	*Le. mesenteroides* subsp. *dextranicum* DSM 20187	2.026	
TRRT08	*Lc. lactis* subsp. *lactis* DSM 20661	2.373	III
	*Lc. lactis* subsp. *lactis* DSM 20481^T^	2.209	
	*Lc. lactis* subsp. *cremoris* DSM 20069^T^	1.848	
TRRT09	*Lc. lactis* subsp. *lactis* DSM 20661	2.283	III
	*Lc. lactis* subsp. *lactis* DSM 20481^T^	2.214	
	*Lc. lactis* subsp. *cremoris* DSM 20069^T^	1.896	
TRRT10	*Lc. lactis* subsp. *lactis* DSM 20661	2.236	III
	*Lc. lactis* subsp. *lactis* DSM 20481^T^	2.198	
	*Lc. lactis* subsp. *cremoris* DSM 20069^T^	1.771	
TRRT11	*La. rhamnosus* CIP A157^T^	2.366	VI
TRRT12	*Lc. lactis* subsp. *lactis* DSM 20661	2.310	III
	*Lc. lactis* subsp. *lactis* DSM 20481^T^	2.150	
	*Lc. lactis* subsp. *cremoris* DSM 20069^T^	1.983	
TRRT13	*Lc. lactis* subsp. *lactis* DSM 20661	2.392	III
	*Lc. lactis* subsp. *lactis* DSM 20481^T^	2.255	
	*Lc. lactis* subsp. *cremoris* DSM 20069^T^	1.988	
TRRT14	*Lc. lactis* subsp. *lactis* DSM 20661	2.371	III
	*Lc. lactis* subsp. *lactis* DSM 20481^T^	2.223	
	*Lc. lactis* subsp. *cremoris* DSM 20069^T^	1.901	
TRRT15	*La. rhamnosus* CIP A157^T^	2.324	VI
TRRT16	*Lc. lactis* subsp. *lactis* DSM 20661	2.456	III
	*Lc. lactis* subsp. *lactis* DSM 20481^T^	2.228	
	*Lc. lactis* subsp. *cremoris* DSM 20069^T^	1.868	
TRRT17	*La. rhamnosus* CIP A157^T^	2.360	VI
TRRT18	*Lc. lactis* subsp. *lactis* DSM 20661	2.521	III
	*Lc. lactis* subsp. *lactis* DSM 20481^T^	2.215	
	*Lc. lactis* subsp. *cremoris* DSM 20069^T^	1.998	
TRRT19	*Lc. lactis* subsp. *lactis* DSM 20661	2.514	III
	*Lc. lactis* subsp. *lactis* DSM 20481^T^	2.196	
	*Lc. lactis* subsp. *cremoris* DSM 20069^T^	1.927	
TRRT20	*Lc. lactis* subsp. *lactis* DSM 20661	2.461	III
	*Lc. lactis* subsp. *lactis* DSM 20481^T^	2.157	
	*Lc. lactis* subsp. *cremoris* DSM 20069^T^	1.992	
TRRT21	*Lc. lactis* subsp. *lactis* DSM 20661	2.538	III
	*Lc. lactis* subsp. *lactis* DSM 20481^T^	2.226	
	*Lc. lactis* subsp. *cremoris* DSM 20069^T^	2.045	
TRRT22	*Lc. lactis* subsp. *lactis* DSM 20661	2.345	III
	*Lc. lactis* subsp. *lactis* DSM 20481^T^	2.286	
	*Lc. lactis* subsp. *cremoris* DSM 20069^T^	1.955	
TRRT23	*La. rhamnosus* CIP A157^T^	2.426	VI
TRRT24	*Lc. lactis* subsp. *lactis* DSM 20661	2.468	III
	*Lc. lactis* subsp. *lactis* DSM 20481^T^	2.243	
	*Lc. lactis* subsp. *cremoris* DSM 20069^T^	2.036	
TRRT25	*La. paracasei* subsp. *paracasei* DSM 20006	2.425	VIIB
	*La. paracasei* subsp. *tolerans* DSM 20258^T^	2.181	
	*La. paracasei* subsp. *paracasei* DSM 5622^T^	2.144	
TRRT26	*La. rhamnosus* CIP A157^T^	2.357	VI
TRRT28	*Le. mesenteroides* subsp. *mesenteroides* DSM 20343^T^	2.358	IB
	*Le. mesenteroides* subsp. *dextranicum* DSM 20187	2.090	
	*Le. mesenteroides* subsp. *cremoris* DSM 20346^T^	2.044	
TRRT30	*La. curvatus* DSM 20499	2.340	V
	*La. curvatus* DSM 20019^T^	2.116	
TRRT31	*La. rhamnosus* CIP A157^T^	2.433	VI
TRRT32	*La. rhamnosus* CIP A157^T^	2.350	VI
TRRT33	*La. paracasei* subsp. *paracasei* DSM 20006	2.435	VIIB
	*La. paracasei* subsp. *paracasei* DSM 5622^T^	2.136	
	*La. paracasei* subsp. *tolerans* DSM 20258^T^	2.103	
TRRT34	*La. curvatus* DSM 20499	2.405	V
	*La. curvatus* DSM 20019^T^	2.166	
TRRT35	*La. rhamnosus* CIP A157^T^	2.367	VI
TRRT36	*Le. mesenteroides* subsp. *mesenteroides* DSM 20343^T^	2.389	IB
	*Le. mesenteroides* subsp. *dextranicum* DSM 20187	2.035	
	*Le. mesenteroides* subsp. *cremoris* DSM 20346^T^	1.985	
TRRT37	*La. paracasei* subsp. *paracasei* DSM 20006	2.448	VIIB
	*La. paracasei* subsp. *tolerans* DSM 20258^T^	2.114	
	*La. paracasei* subsp. *paracasei* DSM 5622^T^	2.000	
TRRT38	*Le. mesenteroides* subsp. *mesenteroides* DSM 20343^T^	2.359	IB
	*Le. mesenteroides* subsp. *dextranicum* DSM 20187	2.131	
	*Le. mesenteroides* subsp. *cremoris* DSM 20346^T^	1.908	
TRRT39	*La. curvatus* DSM 20499	2.234	V
	*La. curvatus* DSM 20019^T^	2.118	
TRRT40	*La. rhamnosus* CIP A157^T^	2.361	VI
TRRT41	*La. rhamnosus* CIP A157^T^	2.354	VI
TRRT42	*Le. mesenteroides* subsp. *mesenteroides* DSM 20343^T^	2.308	IB
	*Le. mesenteroides* subsp. *dextranicum* DSM 20187	2.004	
	*Le. mesenteroides* subsp. *cremoris* DSM 20346^T^	1.982	
TRRT43	*La. paracasei* subsp. *paracasei* DSM 20006	2.362	VIIB
	*La. paracasei* subsp. *paracasei* DSM 5622^T^	2.234	
	*La. paracasei* subsp. *tolerans* DSM 20258^T^	2.182	
TRRT44	*La. rhamnosus* CIP A157^T^	2.405	VI
TRRT46	*La. paracasei* subsp. *paracasei* DSM 20006	2.459	VIIB
	*La. paracasei* subsp. *tolerans* DSM 20258^T^	2.221	
	*La. paracasei* subsp. *paracasei* DSM 5622^T^	2.000	
TRRT47	*Le. mesenteroides* subsp. *mesenteroides* DSM 20343^T^	2.322	IB
	*Le. mesenteroides* subsp. *dextranicum* DSM 20187	2.041	
	*Le. mesenteroides* subsp. *cremoris* DSM 20346^T^	1.864	
TRRT48	*La. paracasei* subsp. *paracasei* DSM 20006	2.242	VIIB
	*La. paracasei* subsp. *tolerans* DSM 20258^T^	1.983	
	*La. paracasei* subsp. *paracasei* DSM 5622^T^	1.658	

**Table 2:** Results of the comparison of the type strains of subspecies from different species of LAB identified in this study obtained with different methodologies.

Strains	Closest Species	Score Values MALDI-TOF	*pheS* Gene Similarity (%)	ANIb (%)	dDDH (%)
*La. plantarum* subsp *plantarum* ATCC 14917^T^ (DSM 20174^T^)	*La. plantarum* subsp. *argentoratensis* DSM 16365^T^	2.424	90.5%	94.9	62.9
*La. paracasei* subsp *paracasei* DSM 5622^T^	*La. paracasei* subsp. *tolerans* DSM 20258^T^	1.846	99.5	97.9	84.9
*Le. mesenteroides* subsp *mesenteroides* ATCC 8293^T^ (DSM 20343^T^)	*Le. mesenteroides* subsp. *cremoris* ATCC 19254^T^ (DSM 20346^T^)	2.456	99.5	98.1	90.9
*Le. mesenteroides* subsp *mesenteroides* ATCC 8293^T^ (DSM 20343^T^)	*Le. mesenteroides* subsp. *dextranicum* DSM 20484^T^	nd	99.2	98.2	91.9
*Le. mesenteroides* subsp *mesenteroides* ATCC 8293^T^ (DSM 20343^T^)	*Le. mesenteroides* subsp. *jonggajibkimchii* DRC1506^T^	nd	99.7	98.4	90.1
*Le. mesenteroides* subsp. *cremoris* ATCC 19254^T^ (DSM 20346^T^)	*Le. mesenteroides* subsp. *dextranicum* DSM 20484^T^	nd	99.7	98.5	91.5
*Le. mesenteroides* subsp. *cremoris* ATCC 19254^T^ (DSM 20346^T^)	*Le. mesenteroides* subsp. *jonggajibkimchii* DRC1506^T^	nd	99.7	98.1	88.5
*Le. mesenteroides* subsp. *dextranicum* DSM 20484^T^	*Le. mesenteroides* subsp. *jonggajibkimchii* DRC1506^T^	nd	99.5	98.4	90.1
*Lc.lactis* subsp *lactis* ATCC 19435^T^ (DSM 20481^T^)	*Lc. lactis* subsp. *cremoris* NBRC 100676^T^ (DSM 20069^T^)	2.174	92.2	86.7	32.7
*Lc.lactis* subsp *lactis* ATCC 19435^T^ (DSM 20481^T^)	*Lc. lactis* subsp. *hordniae* CCUG 32210^T^	nd	99.2	96.7	79.9
*Lc.lactis* subsp *lactis* ATCC 19435^T^ (DSM 20481^T^)	*Lc. lactis* subsp. *tructae* DSM 21502^T^	nd	92.5	86.1	31.7
*Lc. lactis* subsp. *cremoris* NBRC 100676^T^ (DSM 20069^T^)	*Lc. lactis* subsp. *hordniae* CCUG 32210^T^	nd	91.5	86.0	31.4
*Lc. lactis* subsp. *cremoris* NBRC 100676^T^ (DSM 20069^T^)	*Lc. lactis* subsp. *tructae* DSM 21502^T^	nd	98.5	97.5	83.6
*Lc. lactis* subsp. *hordniae* CCUG 32210^T^	*Lc. lactis* subsp. *tructae* DSM 21502^T^	nd	91.8	85.9	31.6

nd: no data because the type strains of some subspecies are not included in the Biotyper 3.0 database.

**Table 3:** Results obtained using MALDI-TOF MS and *pheS* gene analyses.

MALDI-TOF MS Group	Number of Strains	Selected Strains	Closest Taxa	Score Values	*pheS* Gene Similarity (%)
Group IA	6 from “Torta del Casar”	CCDET66,	*Le. mesenteroides* subsp. *mesenteroides* DSM 20343^T^	1.3–2.2	99.2
	1 from “Torta de Trujillo”	CCDET68	*Le. mesenteroides* subsp. *cremoris* DSM 20346^T^	1.6–2.0	99.7
Group IB *	6 from “Torta de Trujillo”	TRRT07,	*Le. mesenteroides* subsp. *mesenteroides* DSM 20343^T^	2.3–2.4	99.7
		TRRT36	*Le. mesenteroides* subsp. *cremoris* DSM 20346^T^	1.8–2.2	99.5
Branch IIA	1 from “Torta del Casar”	CCDET55	*La. diolivorans* DSM 14421^T^	1.9	99.3
Group IIB	9 from “Torta del Casar”	CCDET04, CCDET57	*La. diolivorans* DSM 14421^T^	1.9–2.2	99.3
Group III	13 from “Torta de Trujillo”	TRRT10,	*Lc. lactis* subsp. *lactis* DSM 20481^T^	1.9–2.3	99.5
		TRRT20	*Lc. lactis* subsp. *cremoris* DSM 20069^T^	1.7–2.1	99.0
Group IV	2 from “Torta del Casar”	CCDET07	*La. plantarum* subsp. *plantarum* DSM 20174^T^	2.1–2.3	100
			*La. plantarum* subsp. *argentoratensis* DSM 16365^T^	1.9–2.0	90.8
Group V	4 from “Torta de Trujillo”	TRRT34	*La. curvatus* DSM 20019^T^	2.0–2.2	99.2
Group VI	13 from “Torta de Trujillo”	TRRT03, TRRT32	*La. rhamnosus* CIP A157^T^	2.3–2.4	100
VIIA	8 from “Torta del Casar”	CCDET29	*La. paracasei* subsp. *paracasei* DSM 5622^T^	2.0–2.1	99.5
			*La. paracasei* subsp. *tolerans* DSM 20258^T^	2.0–2.3	99.5
VIIA	8 from “Torta del Casar”	CCDET51	*La. paracasei* subsp. *paracasei* DSM 5622^T^	2.0–2.1	100
			*La. paracasei* subsp. *tolerans* DSM 20258^T^	2.0–2.3	99.7
VIIB	22 from “Torta del Casar”	CCDET16	*La. paracasei* subsp. *paracasei* DSM 5622^T^	1.8–2.3	100
	7 from “Torta de Trujillo”				
			*La. paracasei* subsp. *tolerans* DSM 20258^T^	1.9–2.2	99.7
VIIB	22 from “Torta del Casar”	TRRT46	*La. paracasei* subsp. *paracasei* DSM 5622^T^	1.8-2.3	99.5
	7 from “Torta de Trujillo”		*La. paracasei* subsp. *tolerans* DSM 20258^T^	1.9-2.2	99.5

* These strains presented 100% similarity with respect to *L. mesenteroides* subsp. *jonggajibkimchii* which is not included in Biotyper 3.0.

## Abbreviations

LAB	Lactic acid bacteria
*La.*	*Lactobacillus*
*Le.*	*Leuconostoc*
*Lc.*	*Lactococcus*
